# In vitro effect of microRNA-107 targeting Dkk-1 by regulation of Wnt/β-catenin signaling pathway in osteosarcoma

**DOI:** 10.1097/MD.0000000000007245

**Published:** 2017-07-07

**Authors:** Zhi-Cai Zhang, Jian-Xiang Liu, Zeng-Wu Shao, Fei-Fei Pu, Bai-Chuan Wang, Qiang Wu, Yu-Kun Zhang, Xian-Lin Zeng, Xiao-Dong Guo, Shu-Hua Yang, Tong-Chuan He

**Affiliations:** a Department of Orthopedics, Union Hospital, Tongji Medical College, Huazhong University of Science and Technology, Wuhan, P.R. China; b Molecular Oncology Laboratory, Department of Orthopaedic Surgery, The University of Chicago Medical Center, Chicago, IL.

**Keywords:** β-catenin, Dkk-1, microRNA-107, Osteosarcoma, Signaling pathway, Wnt

## Abstract

**Background:**

The aim of the study was to explore the effects of microRNA-107 (miR-107) by targeting Dkk-1 on osteosarcoma (OS) via the Wnt/β-catenin signaling pathway.

**Methods:**

OS and adjacent tissues were collected from 67 patients diagnosed with OS. Expressions of miR-107, Dkk-1, LRP5, β-catenin, and c-Myc were detected by the quantitative real-time polymerase chain reaction (qRT-PCR) and Western blotting. The dual-luciferase reporter gene assay was performed to observe the relationship between miR-107 and Dkk-1.Transfected cells were divided into different investigating groups designated as Inhibitor, Mimic, siRNA, Inhibitor + siRNA, negative control (NC), and blank groups. qRT-PCR and Western blotting were used to detect expressions of miR-107, Dkk-1, β-catenin, Bcl-2, c-Myc, Caspase-3, and PARP. Cell counting kit-8 (CCK-8), flow cytometry (FCM), colony-formation efficiency (CFE), and subcutaneous tumorigenicity assays were all utilized for to determine cell proliferation, apoptosis, colony-forming, and tumorigenic abilities.

**Results:**

Dkk-1 is the target gene of miR-107. Decreased expressions of miR-107, LRP5, β-catenin, and c-Myc, and increased expressions of Dkk-1 were found in OS tissues. The Mimic and siRNA groups exhibited decreased proliferation rates, colony-forming abilities, and tumorigenicity and increased apoptosis rates, whereas the inhibitor group showed opposite trends when compared to the blank group. On the other hand, expressions of miR-107, LRP5, β-catenin, c-Myc, Caspase-3, and PARP were all elevated in the mimic group, whereas expressions of Dkk-1 and Bcl-2 were reduced; opposite trends were observed in the inhibitor group.

**Conclusion:**

We conclude that miR-107 is likely to inhibit the occurrence and development of OS by down-regulating Dkk-1 via the Wnt/β-catenin signaling pathway, providing us with a new therapeutic target for the treatment of OS.

## 1 Introduction

Osteosarcoma (OS) is a cancerous tumor in bones, ranks as the second most prevalent leading cause of cancer-related death in children and young adults. OS manifests itself as a malignant tumor that occurs approximately 60% in patients under 20 years of age.^[1–3]^ To this day, the pathogenesis of OS has not been discovered and its heterogeneous genetic factors have yet to be elucidated upon.^[4]^ OS is commonly likely to be found in physically active patients, it is hard to diagnose until the tumor progresses to a very late stage.^[5]^ For patients diagnosed with OS, preoperative and postoperative poly-chemotherapy is implemented for treatment. However, this method of treatment is found to be quite ineffective and less than acceptable survival rates especially in pediatrics thus begging for new alternative forms of treatment.^[6]^ The prognosis of 5-year survival for OS patients after limb salvage assisted with chemotherapy was approximately 70%.^[7]^ A previous study conducted in mouse models reported that the abnormal expression of miRNAs may be potential pathologic factors for the development of OS.^[8]^ Therefore, it may be possible that microRNAs (miRNAs) may also have certain implication in the pathogenesis of OS in humans. miRNAs are endogenous, noncoding RNA molecules that functions in RNA silencing and post transcriptional regulation of gene expression. In terms of cancer onset and prognosis, miRNAs can either play a role as an oncogene or tumor suppressor.^[9,10]^ miR-107 is an miRNA located in chromosome 10, that is found to be abnormally expressed in various tumors expressed abnormally in various tumors.^[11–13]^ Evidence has supported that the decreased expression of miR-107 correlates to the progression of cell growth, migration, and invasion in various cancers, including gastric cancer and hepatocellular carcinoma (HCC).^[14,15]^ miR-107 is shown to act as a tumour suppressor and is downregulated in breast cancer, possibly through regulation of its inverted downstream target of *brain-derived neurotrophic factor (BDNF)*.^[16]^ In addition, the EZH2/Wnt/β-Catenin signaling pathway was found to involved in the stumuli and promotion of osteogenic differentiation targetd by another type of miRNA known as miR-101.^[17]^

The Wnt/β-catenin signaling pathway is a pathway that produces an accumulation of β-catenin in the cytoplasm, then the accumulated β-catenin translocate into the nucleus and acts as a transcriptional coactivator of transcription factors.^[18]^ The Wnt/β-catenin signaling pathway is found to play a critical role in the pathogenesis of OS through regulation of bone cell proliferation and differentiation.^[19]^ It was found that the Dickkopf family is able to inhibit the Wnt/β-catenin signaling pathway.^[20]^ In early studies, Dickkopf-1 (Dkk-1) acts as a Wnt inhibitory factor and was reported to produce abnormal bone metabolism.^[19,21]^ Recently, new evidence has a combination of miRNAs dysregulation and the Wnt/β-catenin signaling pathway could contribute to carcinogenesis, cancer metastasis, and drug-resistance.^[22]^ In recent years, several studies have indicated the involvement of miRNAs in tumor progression and metastasis.^[23]^ A better understanding of the regulatory network will provide better insight into miRNA-based therapeutic development, the necessary to combine miRNA and Wnt/β-catenin signaling was urgent. Thus in this paper, we want to investigate the possbile mechanisms of how miR-107, Dkk-1, and Wnt/β-catenin signaling pathway could all contribute to the development and progression of OS cases.

## 2 Materials and methods

### 2.1 Ethical statement

This study was approved by the Ethics Committee of Union Hospital, Tongji Medical College, Huazhong University of Science and Technology. All patients participating have agreed and signed the written informed consent.

### 2.2 Sample collection

From September 2014 to September 2015, 67 patients diagnosed with OS in the Union Hospital, Tongji Medical College, at Huazhong University of Science and Technology, were included in study. Patients tissue containing OS as determined by pathological observations were collected. Age eligibility for this investigation was limited to 40 years old. Patients with secondary OS were excluded from study and all enrolled patients had no history of anti-tumor treatment, such as chemotherapy, immunotherapy, and radiotherapy preoperative during the process of samples collection.

### 2.3 Cell culture, transfection, and establishment of experimental groups

MG-63, U-2-OS, and human osteosarcoma cells (HOS) were purchased from American Type culture Collection (ATCC, Manassas, VA). Cells were cultured in RPMI-1640 containing 10% imported fetal calf serum, 100 U/mL penicillin and 100 mg/L streptomycin in 5% CO_2_ and saturated humidity at 37 °C. Cell passages were digested by 0.125% trypsin containing 0.1% ethylene diamine tetra acetic acid (EDTA). In this investigation, 6 experimental groups were set up: control group, negative control group, miR-107 mimics group, miR-107 inhibitors group, siRNA group, and Inhibitor + siRNA group.

The control group consisted of MG-63, U-2-OS and HOS cells in logarithmic phase were transfected into the blank (without transfection), negative control group (NC group, transfected with an NC sequence; upstream: 5′-UUCUCCGAACGUGUCACGUTT-3′, downstream: 3′-ACGUGACACGUUCGGAGAATT-5′), miR-107 mimics group (Mimic group, upstream: 5′-AGCAGCAUUGUACAGGGCUAUCA-3′, downstream: 3′-AUAGCCCUGUACAAUGCUGCUUU-5′), miR-107 inhibitors group (Inhibitor group, 5′-UGAUAGCCCUGUACAAUGCUGCU-3′), siRNA group (transfected with Dkk-1 siRNA, 5′-TGATAGCCCTGTACAATGCTGCT-3′), and Inhibitor + siRNA group (transfected with miR-107 inhibitors and Dkk-1 siRNA). After a 24-hour cell transfection, cellular RNA was extracted by Trizol method and quantitative real-time polymerase chain reaction (qRT-PCR) was performed to detect mRNA expressions of miR-107 and Dkk-1. Cellular proteins were extracted and the proteins expressions of LRP5, β-catenin, Bcl-2, Caspase-3, PARP, and c-Myc were detected using Western blotting.

### 2.4 Quantitative real-time polymerase chain reaction (qRT-PCR)

The total RNAs were extracted from OS tissues and cell lines. miScript II RT Kit and specific upstream primer of miR-107 were purchased from QIAGEN Company. Total RNA containing miRNA was reverse-transcribed based on the instructions provided by the miScript II RT Kit. PCR amplification was conducted for miR-107 with the utilization of specific upstream primer and universal downstream primer of miR-107. Primers for U6RNA, Dkk-1 and GAPDH were designed using Primer 5.0. qRT-PCR was performed according to the following conditions and procedures: pre-denaturation at 95 °C for 10 minutes, 40 cycles of denaturation at 95 °C for 10 seconds, annealing at 60 °C for 20 seconds and extension at 72 °C for 10 seconds. A temperature of 95 °C was set for 30 seconds to prepare a melting curve. Each specimen added to 3 parallel wells. U6RNA was used as reference for miR-107 and GAPDH as an internal reference for Dkk-1 in order to normalize the readings. All primers are shown in Table 1. The relative quantitative 2^−ΔΔCt^ method was performed to carry out data analysis. The experiment was repeated 3 times.

**Table 1:**
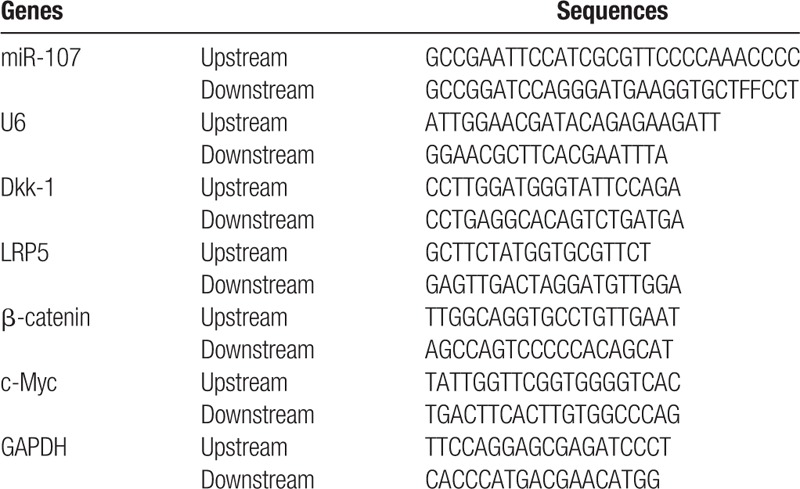
Primers sequences for U6, Dkk-1, and GAPDH.

### 2.5 Western blotting

After washed with PBS, OS cells and OS tissues were lysed in cell lysis buffer supplemented with an adequate addition of protease inhibitor and shaken for 5 minutes at 4 °C, followed by a 10-minute centrifugation at 12,000 rpm at 4 °C. The supernatant was collected and a protein quantification kit was used to detect and obtain the desired protein concentration. Six types of buffers were added and boiled for sample preparation. All 50 μg proteins were then subjected to sodium dodecyl sulfate polyacrylamide gel electrophoresis (SDS-PAGE) system in a wet way which was transferred onto the nitrocellulose membrane. Samples were incubated with primary antibodies (rabbit anti-human antibody Dkk-1, LRP5, β-catenin, c-Myc, Bcl-2, Caspase-3, PARP, and β-actin) overnight, and washed 4 times with TBST (10 mins each time). Then the developing substrate was added for film development. The quantification of protein band was analyzed and processed using LabWorks Image Acquisition and Analysis Software (UVP, Inc., Upland, CA). Experiment was repeated 3 times.

### 2.6 Dual-luciferase reporter gene assay

The targeting relationship between miR-107 and Dkk-1 was predicted using Targetscan database and verified by dual-luciferase reporter gene assay. The full length of 3′UTR region of Dkk-1 amplified gene (Dkk-1 Wt) and PCR products were cloned into the multiple cloning sites downstream of the pmirGLo Luciferase gene (Promega, Madison, WI). Targetscan database was then used to predict the binding site for miR-107 which includes the 756–763 bases of Dkk-1 3’UTR (seed region). The sequence mutation (Dkk-1 Mut) was generated by mutating the sites of seed region. pRL-TK (TaKaRa, Dalian, China) was used as a reference expression vector for railla luciferase. The miR-107 mimics and NC were co-transfected with luciferase reporter vectors (Dkk-1 Wt and Dkk-1 Mut) into MCF-7 cells respectively. The dual-luciferase activity was detected according to the manufacturer's protocol (Promega Corp, Madison, WI). The experiment was repeated 3 times.

### 2.7 Cell counting Kit-8 (CCK-8)

Transfected U-2-OS cells were inoculated in a 96-well plate. Cell counting was conducted at certain time point after cells adhered to walls. The culture medium for U-2-OS cells was first disposed of and 100 μL of fresh culture medium containing 10 μL of CCK-8 reagent was added into the plates. The plates were placed in a CO_2_ incubator for 2 hours before measuring the optical density (OD) value at a wave length setting of 450 nm using a micro-plate reader (Bio-Rad). Cell proliferation rate was calculated with the equation: proliferation rate (%) = OD value in experimental well – OD value of control well)/ OD value of control well × 100%. Six duplicated wells were set for each experiment.

### 2.8 Flow cytometry (FCM)

After cell transfection, U-2-OS cells were harvested and fixed using −20 °C pre-cooled ethanol and stored in a refrigerator at 4 °C overnight. Cells were then centrifuged and washed with cold PBS twice. RNaseA was then added into the cells, and then left in a water-bathed for 30 minutes in the dark along with the addition of Propidiom iodide (PI) for staining. FCM was performed to record cell cycle under red fluorescence. The ratio of cells in G0/G1, S and G2/M phase was calculated. Each experiment was conducted for 3 times.

AnnexinV/*P*I staining was used to detect cell apoptosis. Twenty-four hours after cell transfection, U-2-OS cells were adjusted for cell density to 1 × 10^6^/mL and 0.5 mL of cell suspension (0.5 mL) was extracted and added to centrifuge tube containing 1.25 ul AnnexinV-FITC (NANJING KEYGEN BIOTECH. CO., LTD). This mixture was left at room temperature without light exposure and the centrifuged for 5 minutes at 1000 rpm after15 minutes. After disposing the supernatant cells was resuspended with 0.5 mL pre-cooled binding buffer and added with 10 μL PI. FCM was immediately added (BD) to help detect cell apoptosis. Scatters in the left lower quadrant (Q4) represent healthy cells (FITC-/PI-), the right lower quadrant represents (Q3) early apoptotic cells (FITC + /PI-) and the right upper quadrant (Q2) represents both necrotic and late apoptotic cells (FITC + /PI +). The cell apoptosis rate is calculated by the percentage of early apoptotic cells (Q3) plus the percentage of necrotic and late apoptotic cells (Q2). The experiment was repeated 3 times.

### 2.9 Colony-formation assay

After 24 hours following transfection, cells in the logarithmic phase were collected and trypsinized. The cell suspension was plated in 6-well dishes at an average cell density of 300 cells per well, and left for incubation after a light mix to ensure even distribution of cells. The cells were checked on a daily basis and culture medium changed twice with PBS wash. When an acceptable colony was determined, cells were fixed with 5 mL of 95% methanol for 15 minutes, stained with 10% hematoxylin for 10 minutes and washed with running water and left to dry. After the grid line was marked, acceptable colonies were selected and were counted when more than 50 cells per colony, and the colony-forming rate was calculated as the ratio of the number of colonies to the number of inoculated cells. The experiment was repeated 3 times and the results depended on the average.

### 2.10 Subcutaneous tumorigenicity assays in nude mouse

After 48 hours, the transfected U-2-OS, HOS and MG63 cells were digested, suspended, and counted. 100 μL of cells with an average density of 3 × 10^7^/mL were selected and transferred to a sterile Eppendorf tube (EP) tube. Nine nude mice obtained from Shanghai SLAC Laboratory Animal Co. Ltd. were randomly divided into 3 groups. Each nude muse was injected subcutaneously with approximately 3 × 10^6^ of cells using 1 m sterile syringe. Injections were made to the left (blank group) and right (NC group) dorsal regions of the front foot and the left (Mimic group) and right (Inhibitor group) dorsal regions of the rear foot of nude mice, followed by conventional feeding. Mice were checked every day and the formed tumors were measured with a ruler. Mice were decapitated when the tumors grew to 2 cm in diameter after 2 weeks of monitoring whereby the tumors were removed and photographed.

### 2.11 Statistical analysis

All data was analyzed using SPSS 21.0 software (SPSS Inc, Chicago, IL). Enumeration data was expressed in ratios and percentages, as well as data among groups were compared by the Chi-square test. Measurement data were expressed as mean ± standard deviation (SD), and a *t*-test was performed to compare the average between 2 samples; the comparison among groups was analyzed by the one-way ANOVA method (homogeneity of variance detection was carried out before analysis); pairwise comparison among groups were analyzed by the LSD-*t* test. Spearman's rank correlation analysis was used for correlation analysis. *P* < .05 was considered statistically significant.

## 3 Results

### 3.1 The expressions of miR-107, mRNA and protein expressions of Dkk-1, LRP5, β-catenin, and c-Myc in OS and adjacent tissues

**Figure 1:**
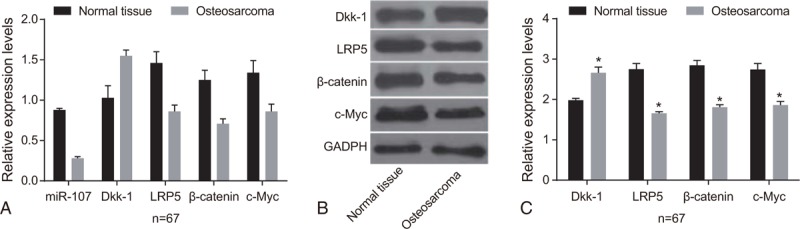
The expressions of miR-107 (A), mRNA (A), protein (B, C) expressions of Dkk-1, LRP5, β-catenin and c-Myc in OS tissues and adjacent tissues detected by qRT-PCR and Western blotting. ^∗^, compared with adjacent normal tissues, *P* < .05; osteosarcoma tissues and adjacent normal tissues were tested (sample numbers = 67 respectively, each sample was detected once), the mean data were obtained from different sample data after the data merged; U6 was used as the reference of miR-107, and GADPH was the reference for other genes. The data was normalized depending on the ratio of the 2. qRT-PCR = quantitative real-time polymerase chain reaction, OS = osteosarcoma.

The results of qRT-PCR and Western blotting demonstrated that mRNA and protein expressions of miR-107, LRP5, β-catenin and c-Myc were lower than adjacent tissue and higher expression of Dkk-1 was in OS tissues compared to adjacent normal tissue (all *P* < .05, Fig. 1). The results of Spearman's rank correlation analysis were shown in Table 2. In OS tissues, miR-107 expression shows a negative correlation compared to Dkk-1 (r = –0.284, *P* = .010). Similarly, a comparison of the expression levels of Dkk-1 and β-catenin showed that significant negative correlation (*r* = –0.281, *P* = .011). On the other hand, miR-107 expression was positively correlated with the expression levels of β-catenin, (*r* = –0.052, *P* > .05). (Table 2)

**Table 2:**
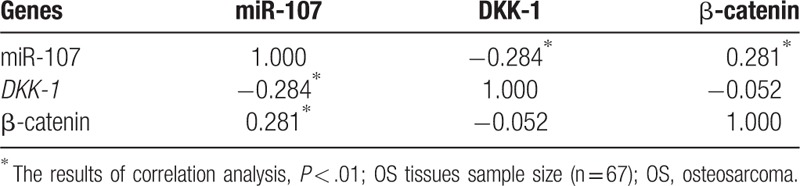
The relationship of miR-107 expression and the expression of DKK-1 and β-catenin in OS tissues.

### 3.2 The expressions of miR-107 and Dkk-1 in MG-63, U-2-OS, and HOS cell lines

**Figure 2:**
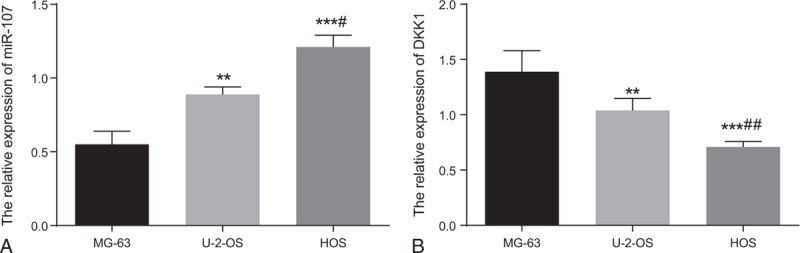
The expressions of miR-107 (A) and Dkk-1 (B) in MG-63, U-2-OS and HOS cell lines. ^∗∗^, compared to the MG-63 cell line, *P* < .01; ^∗∗∗^, compared to the MG-63 cell line, *P* < .001; ^#^, compared to the U-2-OS cell line, *P* < .05; ^##^, compared to the U-2-OS cell line, *P* < .01.

Detections in expression levels in MG-63, U-2-OS, and HOS cell lines showed that the MG-63 cell line had the lowest miR-107 expression whereas the HOS cell line had the highest. On the contrary, the MG-63 cell line had the highest expression levels of Dkk-1 whereas the HOS cell line had the lowest expression (Fig. 2).

### 3.3 Dkk-1 might be a target gene of miR-107

**Figure 3:**
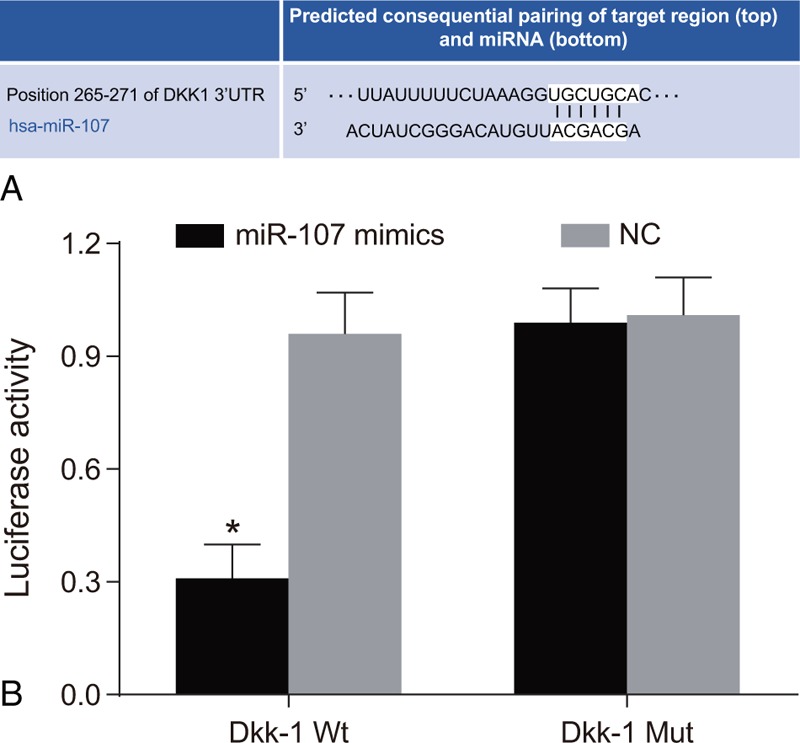
The target relationship between miR-107 and Dkk-1 detected by the dual-luciferase reporter gene assay. (A) Target scan database shows that Dkk-1 might be a potential target gene of miR-107; (B) dual-luciferase reporter gene detected that miR-107 target Dkk-1. The experiment was repeated 3 times.

Targetscan database showed that Dkk-1 was a potential target gene of miR-107 (Fig. 3A). The dual-luciferase reporter gene further verified that the luciferase signal in Dkk-1 Mut co-transfection group decreased by 70% in the Mimic group when compared with other groups (all *P* < .05) (Fig. 3B). However, the luciferase's signal in mutant Dkk-1 Mut group was not significantly decreased with transfection of miR-107 mimics or miR-107 inhibitor and there were no significant differences among groups (all *P* > .05). The results showed that miR-107 binding to 3’UTR of Dkk-1 could inhibit the transcription of Dkk-1 and negatively regulated the expression of Dkk-1. Therefore, Dkk-1 might be a direct target gene of miR-107.

### 3.4 The expression of miR-107, mRNA and protein expressions of Dkk-1, LRP5, β-catenin and c-Myc in MG-63, U-2-OS and HOS cells in 6 groups

**Figure 4:**
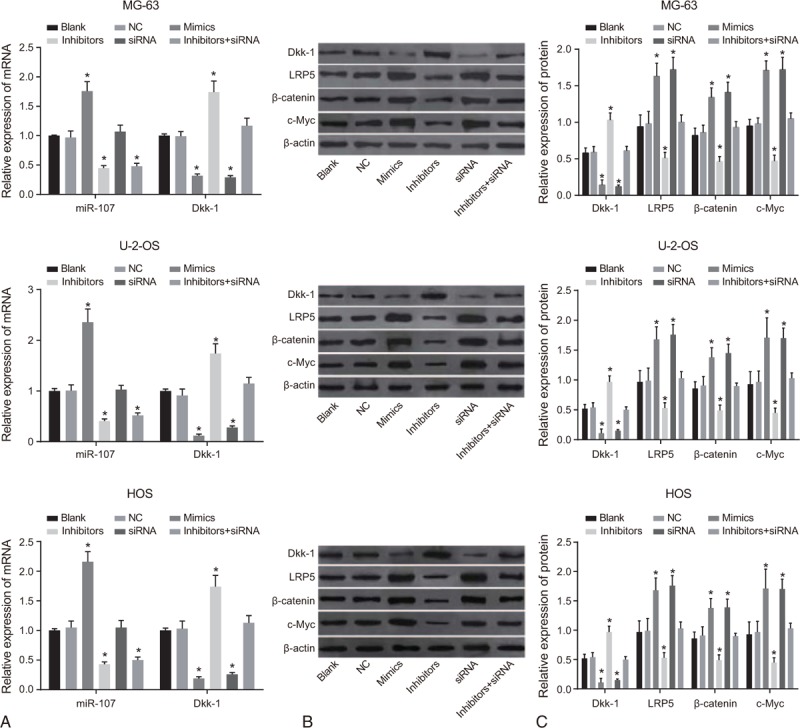
The expression of miR-107 the mRNA (A) and protein (B, C) expressions of Dkk-1, LRP5, β-catenin, and c-Myc in MG-63, U-2-OS, and HOS cells in 6 groups detected by qRT-PCR and Western blotting. ^∗^, compared with the blank group, *P* < .05. The experiment was repeated 3 times.

qRT-PCR demonstrated that the mRNA expressions of miR-107 and Dkk-1 in the blank and NC groups were not significantly different (both *P* > .05). In comparison to the blank group, the mRNA expression of miR-107 in the Mimic group was elevated, whereas that in the inhibitors and inhibitor + siRNA groups was significantly inhibited (all *P* < .05); the mRNA expression of Dkk-1 in the Mimic and siRNA groups was decreased while that in the inhibitor group was notably increased (all *P* < .05) (Fig. 4A), which showed that miR-107 inhibited the expression of Dkk-1.

The result of Western blotting was shown as Fig. 4B and C. There is no significant difference on the expressions of Dkk-1, LRP5, β-catenin, and c-Myc observed in the blank, NC and inhibitor + siRNA groups (all *P* > .05). Compared to the blank group, the expression levels of Dkk-1 were decreased, but expressions of LRP5, β-catenin, and c-Myc were increased in the Mimic group; however, opposite trends were observed in the inhibitor group (all *P* < .05). These showed that in U-2-OS cell line, miR-107 could up-regulate the Wnt/β-catenin signal pathway by targeting and inhibiting the expression of Dkk-1.

### 3.5 Cell proliferation and apoptosis in MG-63, U-2-OS, and HOS cell lines in 6 groups

**Figure 5:**
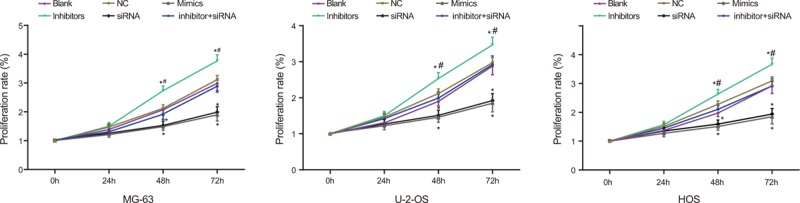
The cell proliferation of MG-63, U-2-OS, and HOS cells among 6 groups. ∗, compared with the blank group, *P* < .05; 6 duplicated well was set for each experiment.

CCK-8 results showed cell proliferation patterns in each group (Fig. 5). The cell proliferation rate of MG-63, U-2-OS, and HOS cells at different time points (0 h, 24 h, 48 h, and 72 h) after transfection between the NC group and the blank group showed no significant difference (all *P* > .05). Twenty-four hours following transfection, cell proliferation rates in the Mimic, siRNA, Inhibitors + siRNA and inhibitor groups were not different to of the blank group (all *P* > .05). After 48 hours, a comparison to the blank group showed significant decreases in proliferation rates in the Mimic and siRNA groups, whereas an increase was found in the inhibitor (both *P* < .05). In contrast, there was no significant difference of the cell proliferation rate in the inhibitor + siRNA group (*P* *>* .05). These results indicated that overexpression of miR-107 and down-regulated Dkk-1 could inhibit the cell proliferation in OS cells and the inhibition of miR-107 could accelerate cell proliferation.

### 3.6 The cell cycle and cell apoptosis in MG-63, U-2-OS, and HOS cells in 6 groups

**Figure 6:**
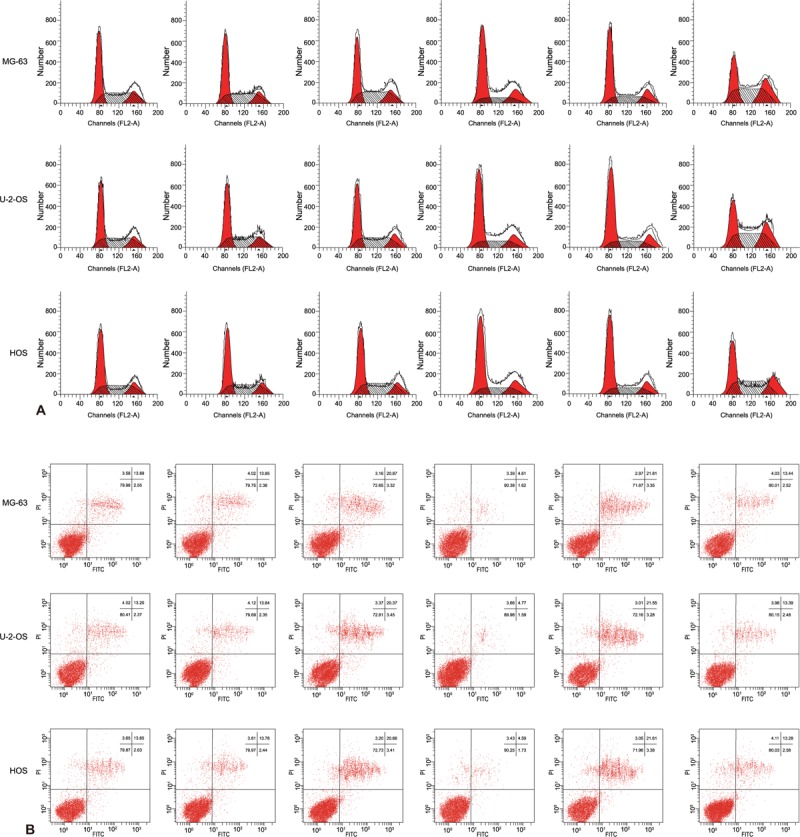
The cell cycle (A) and cell apoptosis (B) of MG-63, U-2-OS, and HOS cells in 6 groups. Note: The experiment was repeated 3 times.

Flow cytometry analysis showed no significant difference in cell cycle to cell apoptosis ratio among the blank, NC, and inhibitor + siRNA groups (all *P* > .05). In comparison to the blank group, the proportion of G0/G1, G2/M phase cells and apoptosis rate in the Mimic and siRNA groups were all increased (all *P* < .05), whereas an increase in the proportion of S and G2/M and a decreased in apoptosis rate was observed in the inhibitor group (all *P* < .05) (Fig. 6). Those results indicated that miR-107 could influence cell progression from G0/G1 phase to S phase.

### 3.7 The protein expressions of Bcl-2, cleavage PARP and cleavage Caspase-3 in 6 groups

**Figure 7:**
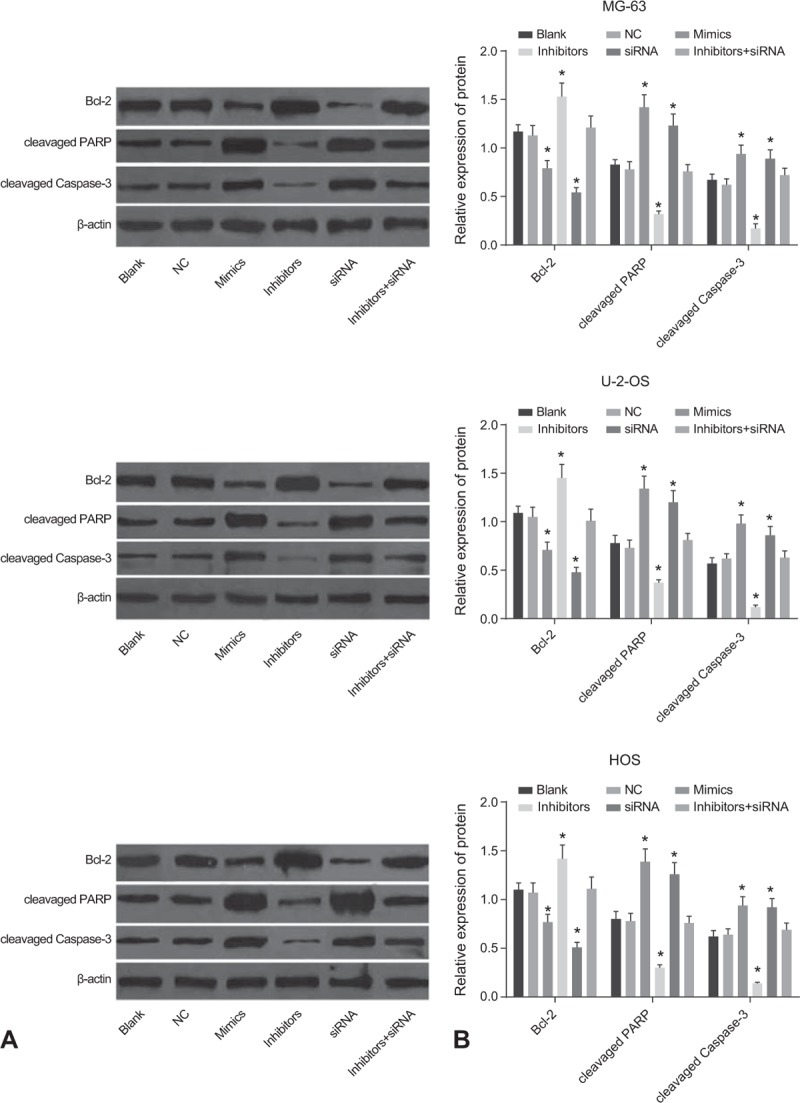
Protein expressions shown as bands (A) and histograms (B) of Bcl2, cleavage PARP, and cleavage of Caspase 3 in different groups. ^∗^, compared with the blank group, *P* < .05; the experiment was repeated 3 times.

The result of Western blotting was shown in Fig. 7. The comparison of the expression levels of Bcl-2, cleavage PARP, and cleavage Caspase-3 in the Blank, NC and inhibitor + siRNA groups indicated no significant difference (all *P* > .05). The expressions of Bcl-2 in the MG-63, U-2-OS and HOS cell lines of the Mimic and siRNA groups were decreased, whereas those of cleavage PARP and cleavage Caspase-3 were significantly increased compared to the blank group (all *P* *<* .05). The opposite trends was observed in the inhibitor group (all *P* > .05). These results indicated that miR-107 could promote cancer cell apoptosis by inhibiting the protein expression of Dkk-1 and down-regulating the protein expression of Bcl-2 in OS cell lines.

### 3.8 The colony-forming ability of MG-63, U-2-OS, and HOS cells in nude mice in 6 groups

**Figure 8:**
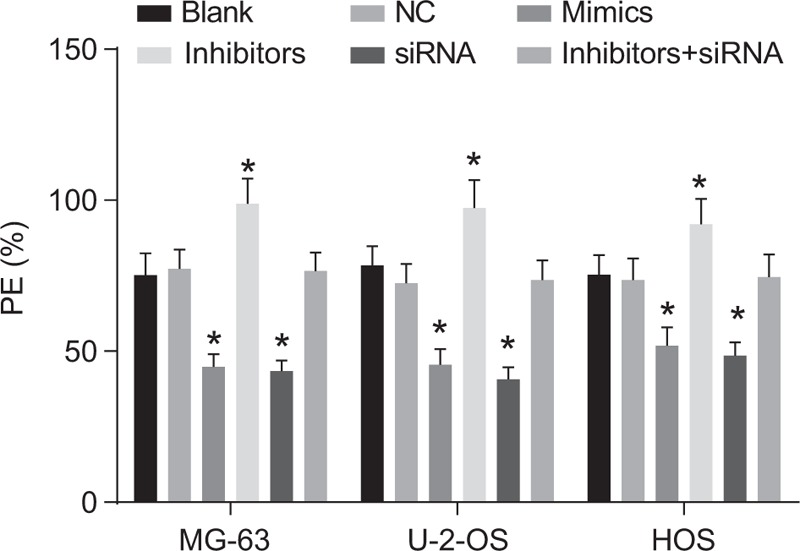
The colony-forming ability of MG-63, U-2-OS, and HOS cells in 6 groups. ^∗^, compared with the blank group, *P* < .05; the experiment was repeated 3 times.

The colony-forming ability of MG-63, U-2-OS, and HOS cells were displayed in Fig. 8. The colony-forming abilities among cells in the NC and inhibitor + siRNA groups indicated no significant difference (all *P* > .05). We also observe that the colony-forming ability in the Mimic and siRNA groups was lowered, whereas that in the inhibitor group was significantly increased when both groups were compared to the blank group (all *P* *<* .05).

### 3.9 Effects of miR-107 on tumor formation of MG-63, U-2-OS and HOS cells in nude mice in 6 groups

**Figure 9:**
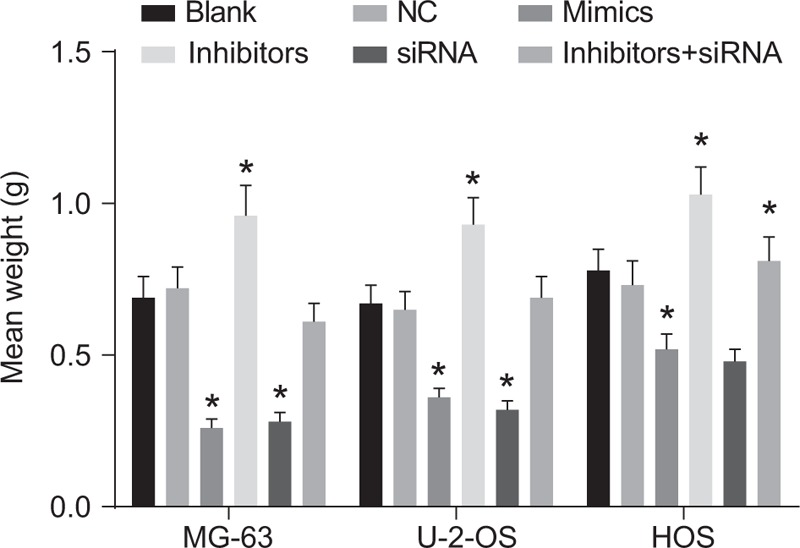
The tumor formation of MG-63, U-2-OS, and HOS cells in nude mice among 6 groups. ^∗^, compared with the blank group, *P* < .05. The experiment was repeated 3 times.

Subcutaneous tumorigenicity assays in nude mice are displayed in Fig. 9. The there was no significant difference in tumor weight among MG-63, U-2-OS, and HOS cells in the NC and inhibitor + siRNA groups (all *P* > .05). Compared to the blank group, tumor weight decreased in the Mimic and siRNA groups, whereas an increase in weight was observed in the inhibitor group (all *P* < .05)

## 4 Discussion

OS is the most frequent and highly malignant primary bone tumor which arises primarily in the metaphysis of the long bones in children and adolescents.^[24]^ OS cells, once abnormally proliferated, are deteriorated into canceration of osteoblasts.^[25]^ Our results found that the OS tissues had decreased expression of miR-107 compared to normal adjacent tissue. Moreover, our in vitro experiments also found that overexpression of miR-107 can inhibit the expression of Dkk-1. This contributes to the upregulation of the expressions of proteins related to the Wnt/β-catenin signaling pathway. Therefore, we hypothesized that in OS, miR-107 serves as a tumor suppressor and negatively targets Dkk-1, which in turn influences the Wnt/β-catenin signal pathway.

Ongoing studies have shown miR-107 to act as a tumor suppressor in other tumors and disease instead of OS, such as breast cancer, non-small lung cancer, and head and neck squamous cell carcinoma.^[26–28]^ Evidence has supported that the expression of miR-107 was significantly downregulated in MCF-7 cells, MDA-MB-231 cells of breast cancer.^[16]^ Several studies also found decreased expression levels of miR-107 in glioma.^[29,30]^ Despite the general findings, it has been reported that miR-107 expression is upregulated in hepatocellular carcinoma and can serve as a contributing factor to the development of HCC.^[31]^ There are also other findings suggest miR-107 is in fact lower in HCC samples compared to adjacent liver tissues.^[15]^ Therefore a lot of research still needs to be done to solidify a definite role of miR-107 in HCC.

In the current study, our results support our hypothesis that miR-107 may act as a tumor suppressor in OS. Dual-luciferase reporter gene assays verified that the miR-107 binding site was located in the 3’UTR region of Dkk-1, suggesting that Dkk-1 is a target gene of miR-107 in OS. As shown in aforementioned results, miR-107 targeting of Dkk-1, leads to an increased expression of miR-107, a downregulation of Dkk-1. This shows us that miR-107 is able to inhibit Dkk-1 gene expression. Some miRNA promoters contain specific binding sites for the cellular signal molecules in Wnt signaling pathway. For example, the promoter activity of *miR-29a* gene could be induced by Wnt3a binding to TCF/TEF.^[32,33]^ This is of particular importance as miR-29a is also discovered to suppress Dkk-1 expression since Dkk-1 is a Wnt signaling pathway inhibitor, downregulation of Dkk-1 will subsequently enhance the Wnt signaling pathway.^[34,35]^ Similarly, another miRNA known as miR-335-5p was also found to be able to down-regulate the expression of Dkk-1, acting as a Wnt antagonist, to inhibit osteoblasts differentiation.^[36]^ Therefore, we concur that miR-107 also has a similar inhibiting mechanism towards the expression of Wnt inhibitors, namely Dkk-1.

This study found lowered expressions of miR-107 and increased expression levels of Dkk-1 in OS tissue. These changes in expressions subsequently weakened the expressions of Wnt/β-catenin signaling pathway-related proteins. The generation of OS cells in response to Dkk-1 was confirmed in a previous study, which suggested that the expression of Dkk-1 can inhibit Wnt/β-catenin signaling by binding to the Wnt receptor complex. As a result of Wnt receptor binding, the Wnt signaling is altered thereby inhibiting osteoblasts differentiation.^[20]^ Dkk-1 inhibits the Wnt signaling pathway though direct binding to lipoprotein receptor-related protein 6 (LRP6), whereas the inhibition on the internalization of LRP6 in turn inhibits glycogen synthase kinase 3β (GSK3β) and activates Wnt downstream signaling.^[37]^ Moreover, the expressions of Dkk-1 was found to be higher at the advanced stages of osteoblasts differentiation when the bone mass and collagen secretion depends on regulated expressions of Wnt/β-catenin signaling pathway-related proteins. In addition, β-catenin could induce the differentiation of mesenchymal cells into osteoblasts, resulting in inhibition on Wnt/β-catenin signaling and apoptosis of osteoblasts.^[38,39]^ Furthermore, the Wnt/β-catenin signaling pathway can enhance the expressions of apoptosis-related factors, such as Bcl-2 and c-Myc.^[20,40]^ Besides, the suppression on Wnt/β-catenin signaling leads to a decrease in the expressions of Bcl-2 and c-Myc, which may disrupt the cell proliferation and apoptosis, and the abnormality in ending cell cycle may cause an abnormal malignant proliferation and tumor.^[41–43]^ Accordingly, our result also found that overexpression of miR-107 can suppress the cell proliferation, while promote cell apoptosis in U-2-OS cells. In this regard, it is speculated that low expression of miR-107 in OS can negatively target Dkk-1, which lead to enhancement of Wnt/β-catenin signaling pathway.

In conclusion, we conclude that the application of miR-107 could be a potential candidate target for the treatment of patients suffering from OS. Better understanding on the mechanisms of miR-107 and how it contributes to OS points us in the new direction for solving the occurrence and progression of multiple diseases.
